# Vitamin E attenuates neurotoxicity induced by deltamethrin in rats

**DOI:** 10.1186/1472-6882-14-458

**Published:** 2014-12-02

**Authors:** Mona K Galal, Abdel Azim A Khalaf, Hanan A Ogaly, Marwa A Ibrahim

**Affiliations:** Department of Forensic Medicine and Toxicology, Faculty of Veterinary Medicine, Cairo University, Giza, Egypt; Department of Biochemistry and Chemistry of Nutrition, Faculty of Veterinary Medicine, Cairo University, Giza, 12211 Egypt

**Keywords:** Deltamethrin, Brain, Apoptosis, Oxidative stress, Vitamin E

## Abstract

**Background:**

The safety of Deltamethrin (DM) has been raised as a point of concern. The current investigation was envisaged to explore the responsiveness of oxidative stress parameters, DNA fragmentation and expression levels of TP53, cycloxygenase 2 (COX2) and cytochrome p4502E1 (CYP2E1) as toxicological endpoint in rats treated with DM. as well as attention was provided to the neuroprotective effect of vitamin E (VE).

**Methods:**

Four different groups of rats were used in this study, group I served as control, group II received DM (0.6 mg/kg BW), group III received both DM plus VE and finally group IV received VE only (200 mg/kg BW). The treatment regimen was extending for one month for all groups and the brain tissues were collected for further analysis.

**Results:**

The obtained results showed a highly statistically significant increase in lipid peroxidation (LPO) content, nitric oxide concentration, and DNA fragmentation percentage and expression level of CYP2E1, TP53 and COX2 genes, in addition statistical significant reduction in total antioxidant capacity in DM treated group as compared to control were detected. Oral administration of VE attenuated the neurotoxic effects of DM through improvement of oxidative status, DNA fragmentation percentage and suppressing the expression level of CYP2E1, TP53 and COX2 genes.

**Conclusion:**

From this study we concluded that VE supplementation has beneficial impacts on DM neurotoxicity in rats through its antioxidant and antiapoptotic properties.

## Background

Although pesticides become instrumental in achieving a significant elevation in crop productivity, they cause serious ecological hazards to the non-target organism [1]. According to WHO [2] roughly three million cases of pesticide poisonings occur annually and an excess of 250,000 deaths worldwide had been reported. The human exposures to pyrethroids were increased since they had been replaced the organophosphorus insecticides [3]. Owing to its low toxicity and its high potency in eradication, DM had become an insecticide of choice in most countries [4]. Although initially thought to be the safest available insecticide, a number of recent reports have been published on its toxicity effect on human, domestics and experimental animals [5, 6]. The direct exposure to DM vapours or consumption of polluted food and water are the most common routes of intoxication [7]. DM induced several pathological changes including inhibition of mitotic index, chromosomal aberrations [8], and induction of histological alterations in several important organs [9]. Recently, attention has been focused on the potential relationship between DM exposure and neurodegenerative diseases [10, 11]. DM creates serious problem because its accumulation in fatty tissue like brain [12]. Whereas the exact mechanism of DM induced neurotoxicity is still poorly understood. Accumulation of DM in body systems increases the reactive oxygen species (ROS) production leading to oxidative stress and apoptotic cell death [5]. The deleterious effects of free radicals accumulation included damage to all macromolecules including proteins, lipids, and nucleic acids. This is believed to be involved in the etiology of many neurodegenerative diseases [12, 13]. Under normal circumstances, the body is endowed with effective antioxidant systems to combat the menace of oxidative stress. Though, in extreme oxidative challenge, such as those observed in pesticide poisoning [14], the body’s antioxidants machineries are overwhelmed. Vitamin E is considered as the most important lipid-soluble antioxidant that protects the brain from oxidative hazard. Many authors reported the neuroprotective influence of VE [15, 16]. VE acts upon cell membranes and has the ability to neutralize compounds which may potentially disrupt membrane stability [17]. Administration of VE decreasing the rate of LPO [11], decreases the autophagy and neuronal death [18], prevents the cytochrome oxidase dysfunction, decreases high energy phosphate compounds and NO in different brain regions as well as it increases the expression level of antiapoptotic gene *Bcl-2* [19] leading to decreased severity of neuronal damage. Although previous studies have already presented the toxicity induced by DM, data on neurodegenerative toxicity are scarce. Moreover, there are very limited studies evaluating the neuroprotective effect of VE against DM toxicity. Therefore; the current study was carried out to investigate the protective influence of VE against DM neurotoxicity through monitoring its effect on oxidative status, DNA fragmentation percentage and the expression level of CYP2E1, TP53and COX2 genes in brain tissue.

## Methods

### Animals

Forty male albino rats, weighing 150–170 g were maintained under standard conditions with free access to food and water. The animals were reared according to the principles of the “Guide for the care and Use of Laboratory Animals” prepared by Beni-Suef University. The Animal care and Use committee of Beni-Suef University approved the study. All efforts were made to minimize animal suffering.

### Chemicals

Deltamethrin (>99% pure) was obtained from KZ pesticide company (Egypt). Vitamin E (α tocopherol) and the rest of chemicals were purchased from Sigma Aldrich chemicals, USA.

### Experimental protocol

After two weeks of acclimation, rats were randomly divided into four equal groups. The group (I) administered corn oil (1 ml/kg BW) and act as control group. The group (II) received oral dose of DM (0.6 mg/kg BW). The group (III) was orally received DM (0.6 mg/kg BW) in combination with VE at a dose of (200 mg/kg BW). The group (IV) was given VE only (200 mg/kg BW). All treatments were continued for 30 days and taken once daily via oral gavage. DM and VE were prepared by dissolving in corn oil. The selected dose of DM was based on previous studies in which 1/10 LD50 induced biochemical alteration in rat without morbidity [20]. Animals were treated with VE in dose that was proved to have neuroprotective effect [21].

### Sampling

All the animals were sacrificed at the end of experiment. Brain was immediately removed and used for further biochemical analysis, DNA fragmentation assay and RNA extraction.

### Biochemical analysis

Brain samples were homogenized in 0.1 M cold phosphate buffer saline (pH 7.4) using Teflon pestle. The homogenates were centrifuged at 14,000 × *g* for 15 min at 4°C. The supernatant was used for measurement of neural LPO expressed as malondialdehyde (MDA) content according to method described by Placer et al. [22], nitric oxide concentration (NO) [23] and total antioxidant capacity (TAC) using commercial kit (purchased from Bio diagnostic company, Egypt).

### DNA fragmentation assay

Apoptotic changes in the brain tissue were evaluated colorimetrically by DNA fragmentation percentage using the diphenylamine (DPA) assay and DNA laddering assay using agarose gel electrophoresis according to the method described by Perandones et al. [24]. Brain samples were homogenized in hypotonic lysis buffer and centrifuged for 15 min at 14,000 rpm. The supernatants containing small DNA fragments were separated; one-half of the volume was used for gel electrophoresis and the other half together with the pellet containing large pieces of DNA were used for quantification of fragmented DNA.

### Isolation of total RNA and real-time PCR (qPCR)

Total RNA was purified from 100 mg of rat brain tissue using Qiagen Rneasy Mini Kit following the manufacturer's protocol. Purity of total RNA was measured spectrophotometeircally (Thermo Scientific, USA). The isolated RNA was reverse transcribed into cDNA and used for PCR with primers specific for CYP2E1, TP53, COX2 and GAPDH (Table 1). The mRNA expression levels of TP53, COX2 and CYP2E1 genes were assessed using real time PCR standardized by co-amplification with the housekeeping gene GAPDH, which served as an internal control. Real-time PCR was done in (color for research Laboratory. Qiagen. Egypt). cDNA was added to a SYBR Green qPCR Master Mix (Qiagen) containing 30 pg/ml of each primer. The cDNA was amplified by 40 cycles of denaturation at 95°C for 15 s, annealing at 60°C for 15 s and extension at 72°C for 45 s. During the first cycle, the 95°C step was extended to 1 min. The GAPDH gene was amplified in the same reaction to serve as the reference gene. Gene expression levels were calculated and determined following the method described by [25].

**Table 1:** **Primers sequences**

Gene	Direction	Primer sequence	Product size	Reference
**CYP2E1**	Forward	TCCAGGTTTGCACCAGACTCT	76 bp	[26]
	Reverse	TCCTCGCTCCTCCTGAGAAG		
**TP53 Exon 7**	Forward	GTG GTA CCG TAT GAG CCA CC	157 bp	[27]
	Reverse	CAA CCT GGC ACA CAG CTT CC		
**COX2**	Forward	AAA GCC TCGTCCAGATGCTA	249 bp	[26]
	Reverse	ATGGTGGCTGTCTTGGTAGG		
**GAPDH**	Forward	ACCACAGTCCATGCCATCAC	460 bp	[28]
	Reverse	TCCACCACCCTGTTG CTGTA		

### Statistical analysis

The data were statistically analyzed by SPSS version 16.0 statistical package. Data expressed as the mean ± SE. Differences between the groups were assessed using one way analysis of variance (ANOVA). Differences were considered statistically significant at P < 0.05.

## Results

### Oxidative stress parameters

The results obtained from Table 2 proved that DM was able to induced oxidative damage. Administration of DM provoked a statistical significant elevation in the level of MDA; the late biomarker of oxidative stress and the good indicator of the degree of LPO in comparing to control one. In the same consequence significant increase in NO concentration was detected. On the other hand a significant reduction in TAC was observed. Oral administration of VE in combination with DM caused a significant reduction in the MDA by 47.67% and NO concentration by 41.24%. Whereas; a significant elevation of TAC by 66.6% was clear in the group III when compared to group II (Table 2). No significant changes were detected between control and VE treated group.

**Table 2:** **Effect of DM treatment on oxidative stress parameters and protective influence of VE supplementation**

Parameters	Group I	Group II	Group III	Group IV
MDA (U/g tissue)	3.17 ± 0.25^b^	10.99 ± 1.16^a^	5.75 ± 0.98^ab^ (47.6%)	2.93 ± 0.84^b^
NO (mmol/L)	2.6 ± 0.4^b^	6.98 ± 0.25^a^	4.1 ± 0.12^ab^ (41.24%)	2.5 ± 0.33^b^
TAC (μ mole/L)	0.289 ± 0.003^b^	0.15 ± 0.0057^a^	0.25 ± 0.007^ab^ (66.6%)	0.29 ± 0.009^b^

Data are expressed as mean ± SEM for 10 animals per group. ^(a)^Represents significant difference from control in the same row while ^(b)^represents significant difference from DM treated group at p < 0.05 in the same row. (% Represent the protective percentage of VE against DM toxicity).

### DNA fragmentation assay

**Figure 1:**
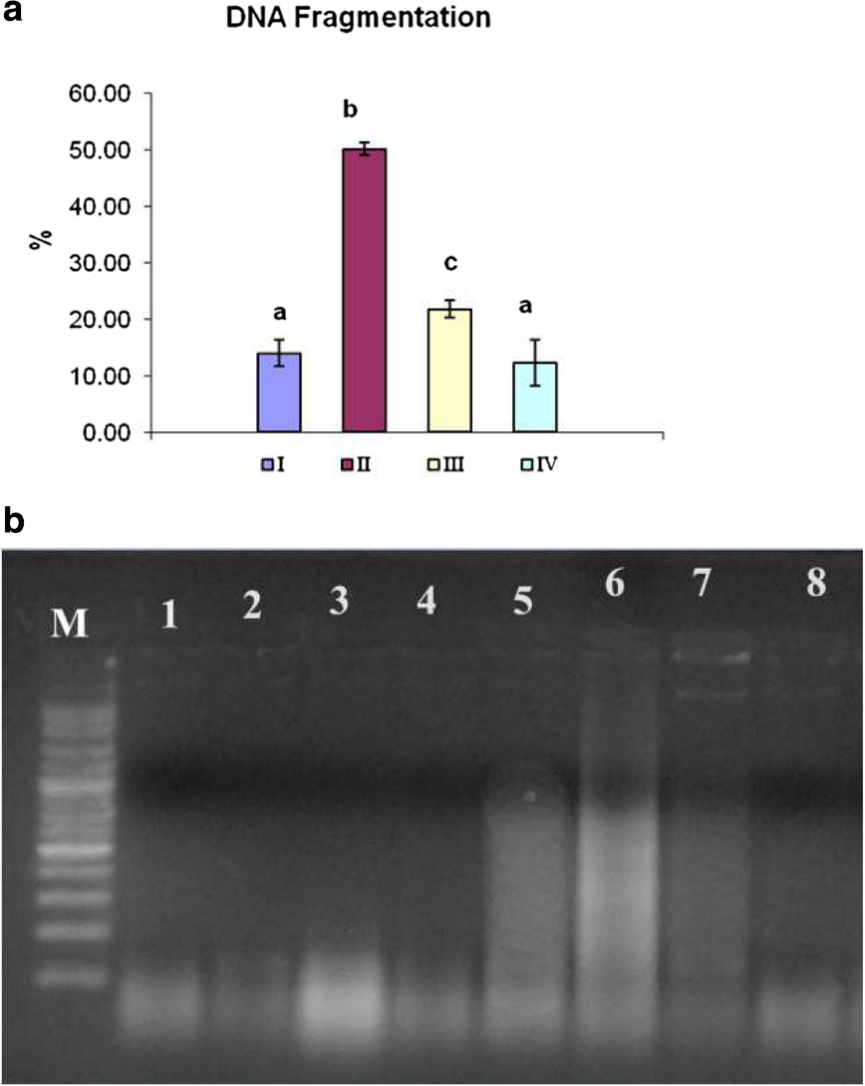
**Protective effect of VE against DM-induced DNA damage in rat’s brain. (a)** DNA fragmentation % by DPA assay; control (I), DM-treated group (II), DM + VE group (III) and VE group (IV). **(b)** The electrophoretic pattern of small DNA fragments on 1.5% agarose gel electrophoresis. Control group (lanes 1, 2) vitamin E control group (lanes 3, 4) DM group (lanes 5, 6) and DM + VE treated group (lanes 7 and 8) M 100 bp DNA marker. Values are expressed as mean ± S.E. Different superscripts letters represents significant difference (p < 0.05).

The adverse effect of DM on DNA was evaluated by measuring the level of genomic DNA fragmentation percentage using the DPA assay (Figure 1a) and detecting DNA laddering on agarose gel electrophoresis (Figure 1b). Compared to the control group, DM induced marked increase in DNA fragmentation level. The VE treated group (III) showed a significant reduction in the DNA fragmentation percentage by 41.6%. The results presented in (Figure 1b) showed marked DNA laddering pattern induced by DM. VE administration proved to reduce the DNA laddering pattern. Lacking of DNA laddering was observed in both group I and IV.

### Gene expression

**Figure 2:**
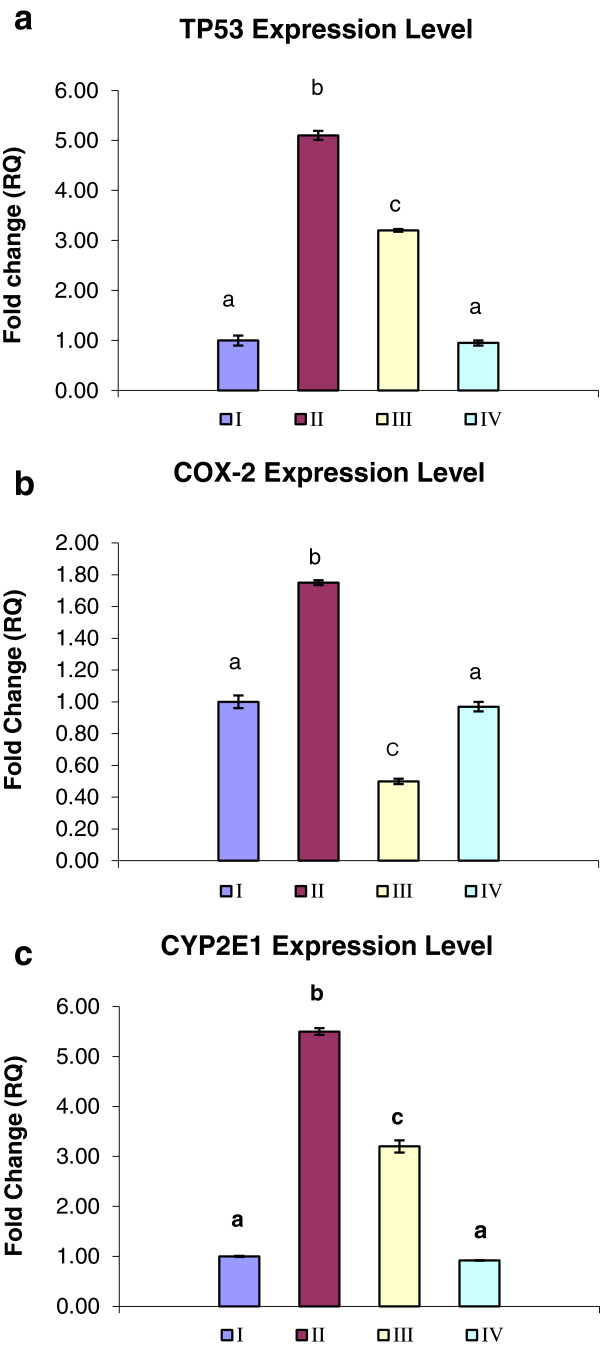
**Real-time PCR Quantitation of mRNA expression level of TP53 (a), COX-2 (b), and CYP2E1 (c): control (I), DM-treated group (II), DM + VE group (III) and VE group (IV).** Values represent fold increases in mRNA level over the control group. GAPDH was used as an invariant internal control for calculating mRNA-fold changes. Values are expressed as mean ± S.E. Different superscripts letters are significantly different (p < 0.05).

Apoptosis, a form of programmed cell death, is tightly regulated by the expression of several genes. In the present study we analyzed the expression of three genes in rat’s brain using quantitative real-time RT-PCR. DM upregulated the expression level for TP53 mRNA to 5.1 fold (Figure 2a). The treatment of rats with VE resulted in reduction in the expression level to 3.2 fold. Furthermore the expression level of COX2 showed 1.75 fold increase in the group II, whereas, the VE ameliorate the adverse effect of DM by decreasing the expression level to the half compared to control (Figure 2b). CYP2E1 showed overexpression after DM intoxication reach to 5.5 fold. Oral administration of VE attenuated the expression to 3.2 folds compared to control (Figure 2c).

## Discussion

In the present investigation we employed advanced methods to uncover the molecular mechanisms of neurotoxicity and apoptotic effect of DM in rat's brain. We assessed the effect of DM on oxidative status, DNA fragmentation percentage and the gene expression level of TP53, COX2 and CYP2E1 and also evaluated the ameliorative role of VE; hence the neurodegenerative studies of DM are limited. ROS are continuously produced inside the mammalian body [29]. Oxidative stress occurred as a consequence of imbalance between the production of free radicals and the antioxidative process leading to instabilities in cellular physiology and apoptotic cell death [29]. In general, pesticides intoxication produces oxidative stress through over production of free radicals and induces tissue LPO in mammals and other organisms [30]. The reduced concentration of TAC and elevation of MDA and NO concentration observed in our present study, suggested that DM causes neuronal damage and the pathogenesis may be through the generation of free radicals and oxidative hazard which certainly play a vital role in the pathogenesis of brain injury. Those results are consistent with the literature [17, 31–34]. The brain tissue contains large amounts of oxidizable substrates such as polyunsaturated fatty acids which are the main target of ROS causing LPO [35]. NO is a potent free radical known to be cytotoxic to neurons and glial cells through its reaction with superoxide and generation of highly reactive radical peroxynitrite [36]. Accumulation of DM in body systems increases the ROS production and leads to depletion of antioxidant parameters which was monitored by significant reduction in TAC (Table 2). The hydrophobic lipophilic nature of DM may be the main causative agent for its accumulation in body systems; ROS production and DNA damage [34]. DNA damage has been proposed as useful parameter for assessing the genotoxic properties of environmental contaminants [37]. DNA fragmentation is considered as a marker and typical characteristic feature of apoptosis [38]. Oral administration of DM had significantly increased DNA damage percentage (Figure 1a). Those results come in the line with data reported by [11, 39]. the oxidative stress induced by DM causes depletion of mitochondrial energy through inhibition of Na, K-ATPases, induction of proteolytic enzymes leading to DNA fragmentation and apoptosis [40]. Also inhibition of ATPases reduces energy supply in neurons leading to neuronal damage [41]. In the same consequence DM caused significant elevation to neutrophiles which play essential role in free radical mediated injury through the extracellular release of superoxide, which is cytotoxic [42]. Our results reported in (Figure 2) are in accordance with other works [5, 43] which suggested that DM induces neurodegeneration and apoptotic cell death in the brain tissue. The ability of DM to cause apoptosis may be contributed to its potential to initiate a series of cell death signaling events that finally lead to DNA fragmentation [11]. According to the results in our study, oral administration of DM increases the mRNA expression of the pre-apoptotic gene TP53 (Figure 2a). Such result is in harmony with those obtained by [38, 43, 44]. Increased ROS production and subsequent DNA damage might be reasons for this overexpression [45]. In oxidative stress p53 protein is activated through multiple posttranslational events including phosphorylation [46]. Activated p53 triggers a number of signaling pathways that may lead to cell cycle arrest and apoptosis [38]. In the CNS, the apoptotic gene (COX2) is expressed under normal conditions [47]. Moreover, it has been demonstrated that COX2 expression is regulated by TP53 gene [48]. According to our finding we detected that, overexpression of COX2 upon DM toxicity. Kim et al. [49] reported that ROS are considered as mediators for COX2 expression. ROS serve as intracellular signals for activation of gene expression through the involvement of specific redox-sensitive signaling pathways and transcription factors [50]. The pervious findings regarding the metabolism of DM revealed that the vast majority of its metabolism occurs in the liver while the extrahepatic metabolism has been reported in other tissues like brain [51]. In rats, the main reaction involved in DM detoxification is ester cleavage, by cytochrome P450 (CYP) enzymes and carboxyesterase action [52]. CYP2E1 is a widely distributed isoform in the brain and is the main inducible CYP against environmental chemicals [53]. The regulation of CYP2E1 expression was occurred through several mechanisms such as protein stabilization and increased translational efficiency which implicated by xenobiotics [54]. Although CYP2E1 plays important role in neuronal detoxification [55], its induction in the brain could increase the risk of neurotoxicity. CYP2E1 bioactivities several cytotoxins to their reactive intermediates and generate ROS and substrate-derived radicals especially when it is induced [56]. Those may be the main cause for CYP2E1 overexpression after DM intoxication (Figure 2c). Those results were in the line with that reported by [53, 57]. In the current study, the oral administration of VE (200 mg/kg) as antioxidant proved to reduce the neurotoxicity induced by DM through statistical significant reduction for MDA, NO concentration and elevation of the TAC (Table 2). Moreover, VE significantly reduced the DNA fragmentation percentage (Figure 1a) and DNA laddering pattern (Figure 1b). In addition VE attenuated the overexpression of TP53 by 37.2%, COX2 by 50% and CYP2E1 by 41.8%, in compared to DM treated group (Figure 2). Oxidative stress plays a major role in DM induced toxicity. Vitamin E is a potent antioxidant which is reported to ameliorate the effect of many known chemotherapeutic agents and pesticides as well. Many previous literatures showed the protective effect of VE against DM intoxication [58–60]. Vitamin E plays a central role against oxidative damage in neuronal tissue as it is the major lipid-soluble chain breaking antioxidant which effectively protects against neuronal damage [19]. VE maintained the activities of membrane bound enzymes at near normal values and thus preserving mitochondrial membrane integrity and protected enzyme activities from oxidation by free radicals [61]. The protective effect of VE against DNA damage comes in the line with the result reported by [62]. VE play an important role in prevention of neuronal death not only by elimination of ROS through its antioxidant ability, but also through non-antioxidant properties like influencing cellular signaling and transcriptional control [63, 64].

## Conclusion

In the current investigation we can concluded that, DM is a potential neurotoxic pesticide, it induced degeneration and apoptotic cell death in rat's brain. On the other hand, VE is proved to be a potent antioxidant which ameliorates the adverse effects triggered by DM.
